# Carbazole Substituted BODIPYs

**DOI:** 10.3389/fchem.2019.00841

**Published:** 2019-12-10

**Authors:** Iti Gupta, Praseetha E. Kesavan

**Affiliations:** Department of Chemistry, Indian Institute of Technology Gandhinagar, Gandhinagar, India

**Keywords:** BODIPY, carbazole, fluorescence, absorption, dyes

## Abstract

Difluoroboron-dipyrromethenes (BODIPYs) are highly popular fluorescent dyes with applications as NIR probes for bioimaging, fluorescent tags/sensors and as photosensitizers in cancer therapy and organic photovoltaics. This review concentrates on the synthesis and spectral properties of BODIPY dyes conjugated with carbazole heterocycle. The carbazole is an electron rich tricyclic compound and due to its excellent electronic properties, it is extensively used in the production of electroluminescent materials and polymers. This review highlights the recent progress made on the series of BODIPY derivatives containing carbazole ring at *alpha, beta*, and *meso*-positions of the BODIPY skeleton. Carbazole based hybrid BODIPYs, carbazole linked aza-BODIPYs and carbazole-fused BODIPYs are also discussed.

## Introduction

**Figure 1:**
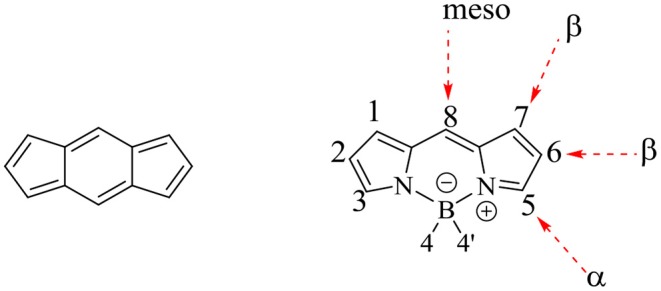
s-indacene and basic BODIPY core structure.

Certain organic or inorganic molecules can act as fluorophores; and they can re-emit the light upon irradiation with the light source. The fluorescent organic dyes have been extensively used in the wide range of applications such as: biomolecular labels (Celli et al., 2010; Kowada and Kikuchi, 2015), chemosensors (Wu et al., 2015), energy transfer cassettes (Fan et al., 2013), organic light emitting diodes (Zampetti et al., 2017), dye-sensitized solar cells (Klfout et al., 2017), etc. Among the highly fluorescent organic molecules reported in the literature, the dyes based on 4,4-difluoro-4-bora-3α,4α-diaza-s-indacene (difluoroboron dipyrromethene, abbreviated as BODIPY, Figure 1); show possibly the highest potential and have become enormously popular in recent times. Although, Treibs and Kreuzer first reported these molecules in 1968 (Treibs and Kreuzer, 1968); the field was not developed much till 1980. In the 1980s, researchers reported potential use of these dyes for biological labeling (Vedamalai et al., 2018). After that, there was a clear rise in the number of reports on BODIPY dyes; making them hugely popular among chemists and biologists to develop BODIPY based fluorescent sensors (Vedamalai et al., 2016, 2018), bioimaging agents (Kesavan et al., 2019), and photosensitizers for PDT (Kamkaew et al., 2013; Zheng et al., 2018).

By mid of the 1990s BODIPY's potential applications in the area of biological sciences (Vedamalai and Gupta, 2018) and materials sciences were fully recognized and research reports in this area tremendously increased (Ulrich et al., 2008). These molecules showed remarkable properties like sharp absorption and emission, large molar absorption coefficients, high fluorescence quantum yields and high photo-stability. Thus, this group of fluorescent dyes meet the criteria for a good fluorophore; they exhibit enormous synthetic variations and versatile applications (Loudet and Burgess, 2007; Ziessel et al., 2007; Kolemen and Akkaya, 2018). Understanding the photophysical properties of these systems (Lu et al., 2014) is of principal importance, not only because of the intrinsic potential applications but, also in the design of new dyes with specific properties. The main synthetic advantage of BODIPY dye is that, the unique structure of dye skeleton provides eight positions which can be easily functionalized to fine-tune their electronic properties (Lakshmi et al., 2015, 2016). There are excellent reviews available on the BODIPYs based on the different applications, such as: fluorescent NIR probes (Yuan et al., 2013), sensitizers for PDT (Kamkaew et al., 2013), organic materials for photovoltaics (Bessette and Hanan, 2014), D-A type systems with focus on energy/electron transfer (Loudet and Burgess, 2007), fluorescent sensors (Boens et al., 2012; Ni and Wu, 2014), BODIPY based multi-chromophore cassettes (Ziessel et al., 2007), etc. This review presents the structural diversity of the carbazole-BODIPY conjugates, with the emphasis on the effect of the substitution of carbazole heterocycle on the optical properties of the BODIPYs.

Carbazole is a well-known heterocyclic aromatic system. The aromatic nature of carbazole makes it chemically and thermally stable; and the ring can be easily functionalized at different positions. Carbazole and its derivatives are electron rich compounds and they exhibit good absorption and emission properties (Li et al., 2004; Barberis and Mikroyannidis, 2006; Mudadu et al., 2008). Also, due to their excellent photoluminescence and hole-transport property; these systems are used for various applications in photovoltaic systems and OLEDs. They are also employed as photosensitizers (Promarak et al., 2008; Wang et al., 2008; Tang et al., 2010) in DSSCs. Carbazole derivatives are also known for their anti-microbial, anti-tumor properties and as bioimaging agents (Głuszyńska, 2015).

In recent years, the reports on carbazole substituted BODIPYs and porphyrinoids (Das and Gupta, 2019) have significantly increased. It is observed that, the presence of electron rich carbazole moiety can alter the absorption and emission properties of BODIPYs; which depend on the position and kind of linkage through which carbazole is attached on the BODIPY skeleton. In this review, we present the overview of synthetic strategies used to prepared various kinds of carbazole substituted BODIPYs; also, the change in the electronic properties due to substitution and their applications are discussed.

### *Beta*-Substituted BODIPYs

Main advantage of BODPY core is that, the three available positions (α, β, and *meso*) are prone to derivetisation (Figure 1). But the feasibility of substitution is highly depended on the other functional groups, already present on the BODIPY core. The substitution of electron rich groups at the *beta*-positions is expected to enhancethe electronic communication between the BODIPY core and the substituents.

**Scheme 1:**
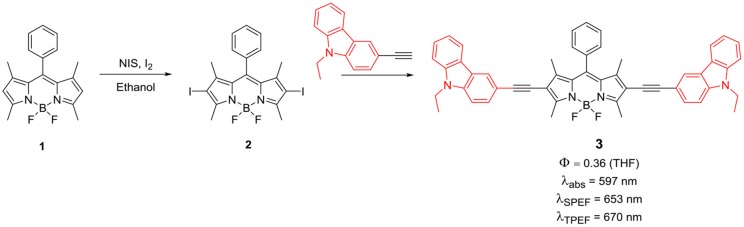
BODIPY–carbazole conjugates inked via ethyne bridges.

In 2009, Zhang et al. reported the synthesis of BODIPY **3** (Scheme 1); the key precursor **2** was coupled with 9-ethyl-3-(prop-1-ynyl)-9*H*-carbazole *via* Pd-catalyzed Sonogashira reaction. The ethynyl linkages present in this molecule helped to show efficient ICT process. The absorption and emission maxima of BODIPY **3** were very much red shifted (Scheme 1) as compared to the parent *meso*-tetraphenyl BODIPY, which reflected the effect of carbazole ring linked via rigid ethyne linker to the boron-dipyrrin core. The linear D-π-D type structure resulted in the extended conjugation along with efficient ICT process, which made this molecule to exhibit two-photon absorption properties (Zhang et al., 2009). As a result, this compound showed a sharp emission peak around 670 nm with reasonable quantum yield. This emission was attributed to two-photon emission fluorescence (TPEF). Since this emission wavelength fall in human body's therapeutic window (650–800 nm), this molecule has potential in bioimaging applications.

**Scheme 2:**
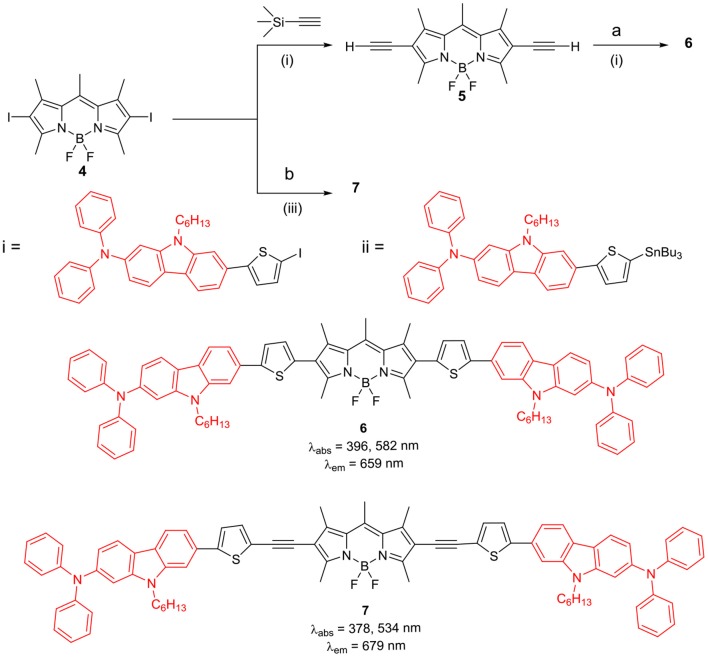
BODIPYs linked to carbazole derivatives with thiophene spacer. (i) Pd(PPh_3_)_2_Cl_2_, CuI, THF, Et_3_N, TBAF, THF; (ii) Pd(PPh_3_)_2_Cl_2_, CuI, THF, diisopropylamine; (iii) Pd(PPh_3_)_2_Cl_2_, PPh_3_, DMF.

**Scheme 3:**
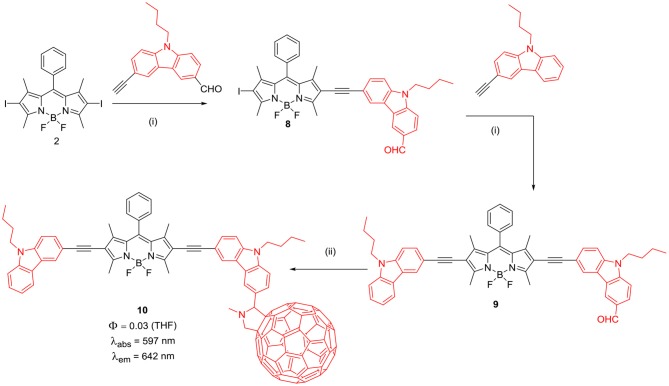
Synthesis of BODIPY linked via ethyne linkage to carbazole and C_60_. (i) PdCl_2_(PPh_3_)_2_, PPh_3_, CuI, NEt_3_, THF, argon atmosphere, 60°C, 6 h; (ii) sarcosine, C_60_, toluene, reflux.

**Chart 1:**
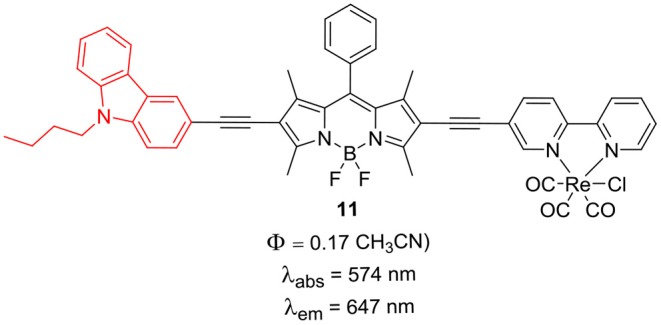
Carbazole BODIPY with rhenium metal complex.

The BODIPYs **6** and **7** linked to carbazole via thiophene spacer were used for organic photovoltaic (OPV) applications (Lin et al., 2012). The target BODIPYs **6** and **7** were synthesized by Stille and/or Sonogashira coupling reactions between precursors BODIPY **4** and **5** with the appropriate stannyl functionalized carbazole derivative (Scheme 2). The studies showed that, insertion of an alkyne moiety renders flexibility between the donor carbazole and acceptor BODIPY core, which is beneficial for light harvesting. The large Stokes shift displayed by BODIPYs **6** and **7** suggests that, the excited state of the molecules have a more planar conformation, which is not favorable for solar cell applications. Both the molecules **6** and **7** showed reasonable light to current conversion efficiency of 1.8 and 2.6%, respectively. Zhao and co-workers reported BODIPYs **9** and **10** where, one *beta*-position is substituted with carbazole ring and the other *beta*-position is linked to C_60_ (Scheme 3) (Yang et al., 2012) or rhenium metal complex (Chart 1). In BODIPYs **9** and **10**, the electron rich carbazole derivative is attached through rigid ethynyl bond by Pd(0) catalyzed Sonogashira coupling. The carbazole derivative linked to the BODIPY unit, is acting as a light-harvesting antenna system. In compound **10**, intramolecular energy transfer was observed from the BODIPY based singlet excited state to the singlet excited state localized on the C60 unit.

The intrinsic intersystem crossing resulted in the triplet-excited state of the C_60_ in the absence of a heavy atom. The dyad **10** showed TTA up-conversion with quantum yield up to 2.9% (Yang et al., 2012). Complex **11** showed much weaker TTA up-conversion, which can be assigned to the weak absorption of **11** at the excitation wavelength and less efficient ISC (Yi et al., 2013).

**Scheme 4:**
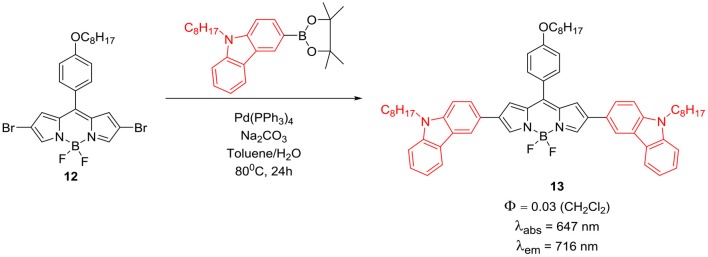
Synthesis of directly linked carbazole-BODIPY system.

**Scheme 5:**
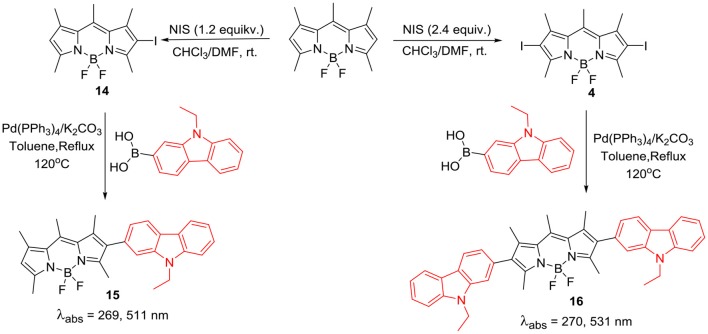
Synthesis of 2,6-direct linked carbazole BODIPY dyad and triad.

Direct substitution at the *beta*-positions of BODIPYs (Scheme 4) through Suzuki coupling between boronic ester derivative of carbazole and the 2,6-dibromo substituted BODIPY **12** is another method to incorporate carbazole ring on the BODIPY core. The pronounced effect of substitution of carbazole rings on the *beta*-position of the BODIPY, reflected in the increased absorption efficiency from 300 to 900 nm; thus molecule **13** acted as a panchromatic dye. This molecule showed an excellent red-shift in its emission and had high thermal stability. Photovoltaic performance studies showed that, by further engineering the molecular structure and optimization of the morphology; this type of dyes can become potential candidates for the efficient organic solar cell materials (Liao et al., 2016). Wanwong et al. reported the application of *beta*-carbazole substituted BODIPY dyad and triad (Scheme 5; **15** and **16**) as field effect transistors (FET). Though these derivatives provided moderate performance, modification of this structure may help to develop better dye with better performance (Wanwong et al., 2018).

**Scheme 6:**
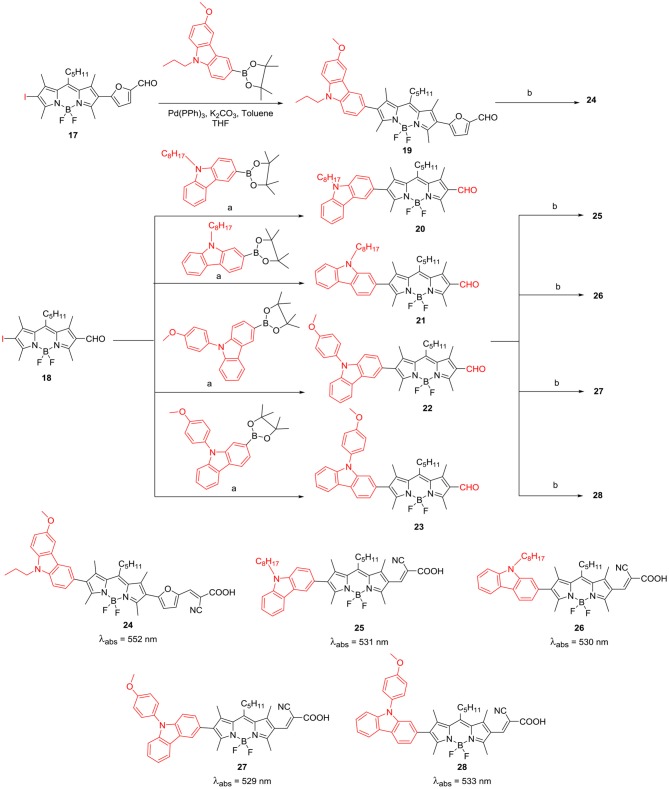
*beta*-carbazole substituted BODIPYs for DSSC application; a: Pd(PPh_3_)_4_, K_2_CO_3_, toluene/THF; b: piperidine, CNCH_2_COOH, DCM/ACN.

Electron rich carbazole ring acts as very good electron donor and its derivatives exhibit photorefractive and hole-trasnport properties; thus they are polupar constituents of electroluminescent materials. Mao et al. reported application of *beta*-substituted BODIPYs, having D-A-π-A system (Scheme 6) for DSSC applications. Incorporation of the extra acceptor in between the donor moiety and π-conjugating unit decreases the HOMO-LUMO energy gap, and as a result, these BODIPYs can show an efficient photoinduced electron transfer from the donor to the BODIPY acceptor unit; which is linked to the anchor group at the opposite end (Mao et al., 2017).

**Chart 2:**
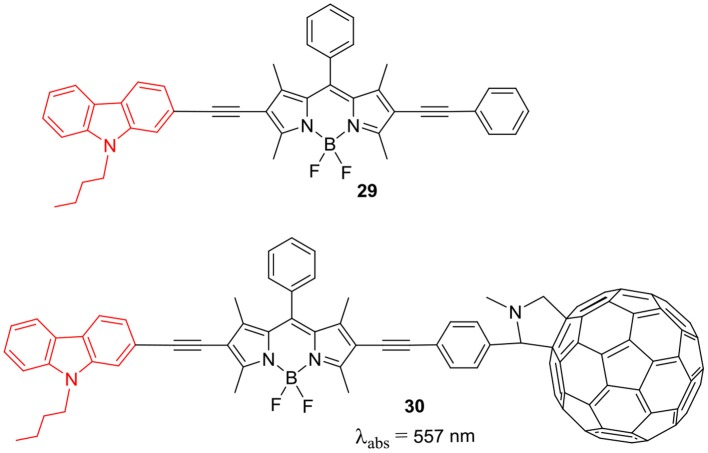
Carbazole-BODIPY-C60 triad as triplet sensitizer.

BODIPY **24** with cyanoacetic acid anchoring group was used as a photosensitizer for DSSC; this linear system showed reasonable PCE efficiency of 3.1%. A series of *beta*-substituted BODIPYs **25–28** having D-π-A system were constructed for DSSC application (Scheme 6). The *N*-alkyl carbazole ring served as donor and BODIPY core linked to cyanoacetic acid was the acceptor unit (Liao et al., 2017b). The DSSC analysis revealed that photosensitizers **26** and **28**, having 2-carbazolyl substituent at the *beta*-position showed better *J*_sc_ than BODIPYs **25** and **27** where carbazole is substituted through 3-position and it was reflected in their overall efficiency (Liao et al., 2017b). BODIPY derivatives having heavy atoms such as: bromo, iodo groups on the dipyrrin core, have been used as triplet sensitizers. Such metal-free triplet sensitizers can be effectively used for singlet-oxygen generation, PDT agents and triplet-triplet annihilation upconversion (TTA-UC). Wei et al. (2017) have prepared BODIPY based organic photosensitizers connected to C_60_ and *N*-butylcarbazole at the β-positions **29** and **30** (Chart 2). As per the report, C_60_ has high ISC (inter system crossing) efficiency but weak absoprtion in the visible region of the solar spectrum. Thus, linking of C_60_ to the carbazole substiuted BODIPY can be useful to effectually populate the triplet excited state of the C_60_, which in turn can transfer the energy to perylene acceptor. The calculated TTA-UC quantum yield was 4.9 for the carbazole-BODIPY—C_60_ triad **30** shown in Chart 2 (Wei et al., 2017).

### *Alpha*-Substituted BODIPYs

There are two alpha positions on the BODIPY skeleton which are available for derivatization after various reactions such as: nucleophilic or electrophilic substitution, cross-coupling reactions, and Knoevenagel condensation (Wood and Thompson, 2007), etc.

**Scheme 7:**
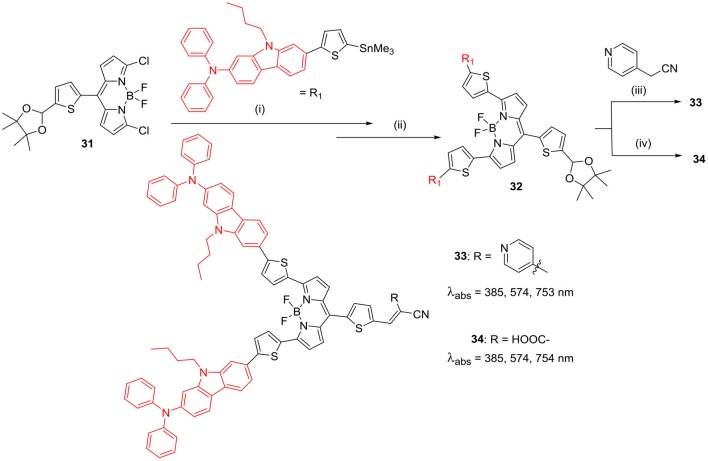
Synthesis of 3,5-carbazole substituted BODIPY for DSSC. (i) Pd(PPh_3_)_4_, toluene; (ii) 2M HCl, THF; (iii) NEt_3_, CH_2_Cl_2_; (iv) Cyanoacetic acid, piperidine, CHCl_3_/CH_3_CN

Ooyama et al. recently reported a new strategy to develop effective BODIPY based sensitisers possessing a good light harvesting efficiency (LHE) in the range of visible light to NIR region. They developed (D)_2_-π-A type BODIPYs **33** and **34** (Scheme 7) having pyridyl and cyanoacrylic acid groups, respectively. The electron-withdrawing anchoring group (pyridyl/cyanoacetic acid) helps to bind the BODIPY on the titanium dioxide layer for photovoltaic application. The presence of strong electron-donating units of 9-butyl-*N,N*-phenyl-7-(thiophen-2-yl)-9*H*-carbazol-2-amine at *alpha*-positions of the BODIPY core, helped to obtain strong and broad absorption band ranging from 600 to 850 nm. Also, these molecules showed high LHE in the range of visible light to NIR region. Though these molecules showed good photophysical properties; these BODIPY dyes showed low photovoltaic performance in DSSC studies, which was attributed to the low lying LUMO levels (Ooyama et al., 2017).

**Scheme 8:**
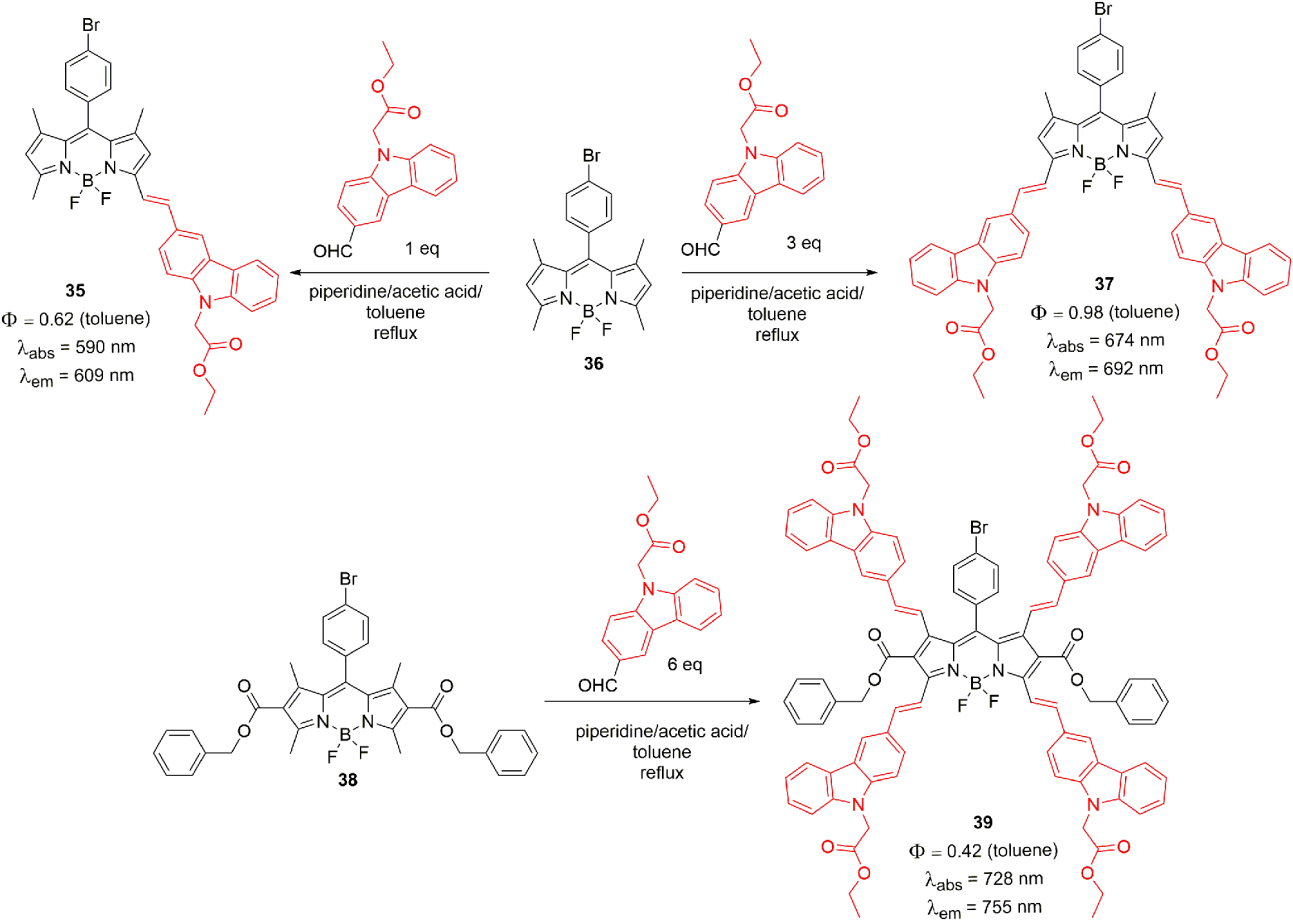
Synthesis of star-shaped BODIPY derivatives.

Knoevenagel condensation is another method to introduce carbazole unit on the BODIPY core. Zhang et al. reported mono-, di- and tetra-styryl carbazole substituted BODIPYs (**35**, **38**, and **39**). The Knoevenagel condensation between methyl-2-(2-formyl-9H-crbazole-9-yl)acetate with BODIPY **35** (Scheme 8) produced target styryl BODIPYs **36**, **37**, and **38** in good yields. The extended π-conjugation converted the simple BODIPYs to NIR dyes with strong absorption maxima in between the 600 and 727 nm range. Their corresponding emission maxima were also considerably red shifted; which reflected the increased conjugation between the carbazole units and the BODIPY core. The BODIPY **39** with four styryl-carbazole unit showed the highest PCE of 2.7% (Zhang et al., 2015).

**Chart 3:**
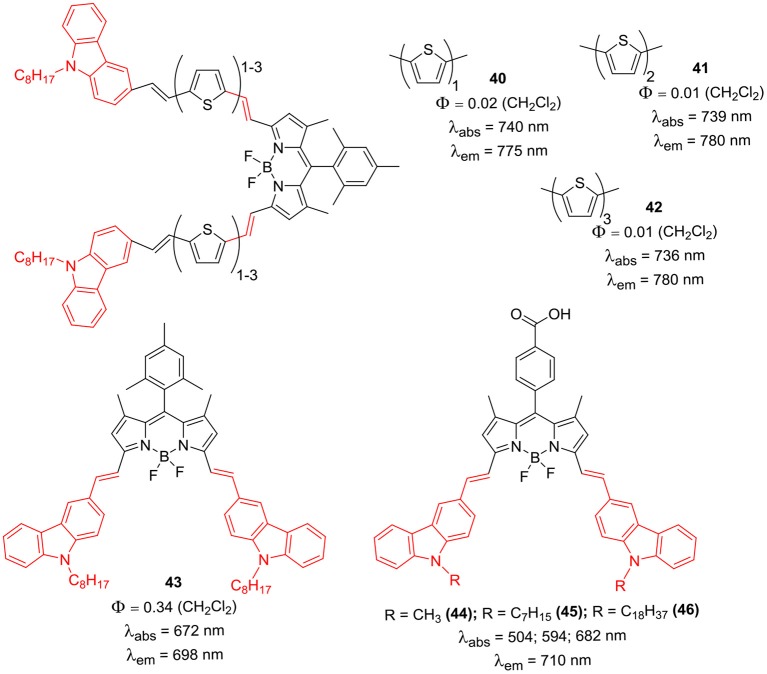
α-styryl linked carbazole BODIPYs.

The synthesis of *di*-styryl BODIPY based D-A and D-π-A systems (**40–44**) was reported, where thiophene served as π-linker between the *N*-alkylcarbazole unit and the BODIPY core (Chart 3) (Brzeczek et al., 2016; Kurowska et al., 2018). The target BODIPYs were synthesized through multistep synthetic procedure; firstly the precursor 1,3,5,7-tetramethyl-8-mesityl BODIPY was prepared by the conventional synthetic protocol. Microwave assisted Knoevenagel condensation of the precursor BODIPY with appropriate carbazole derivative afforded target BODIPYs **40–44** (Chart 3). The carbazole substituion at *alpha*-positions of the BODIPY core has prominent effect on the electronic properties of the dyes; the target compounds **40–44** showed markdly red shifted absorption (736–740 nm) and emission maxima (775–780 nm). Particularly, the BODIPY–carbazole conjugates with single thiophene linker showed highly red-shifted absorption and emission spectra. On increasing the number of thiophene linkers, the effect of carbazole donor on the BODIPY acceptor was diminished; the quenched emission was attributed to the stronger push-pull effect for systems with elongated conjugation framework.

Followed by this work, Cheema et al. studied the application of 3,5-di-styryl BODIPYs having *N*-alkyl carbazole units (**45–46**) for DSSC application (Chart 3). Carboxylic acid group on the *meso*-phenyl ring of the BODIPY acted as anchor group (Cheema et al., 2016). It was revealed that, the alkyl substitution did not change the position of absorption and emission maxima; but the intensity of these bands was altered. With the increases in the length of *N*-alkyl chain, the intensity of the lower energy absorption band decreased. DSSC performance for the dyes was much less than expected, which was attributed to the aggregation related losses (Cheema et al., 2016).

**Scheme 9:**
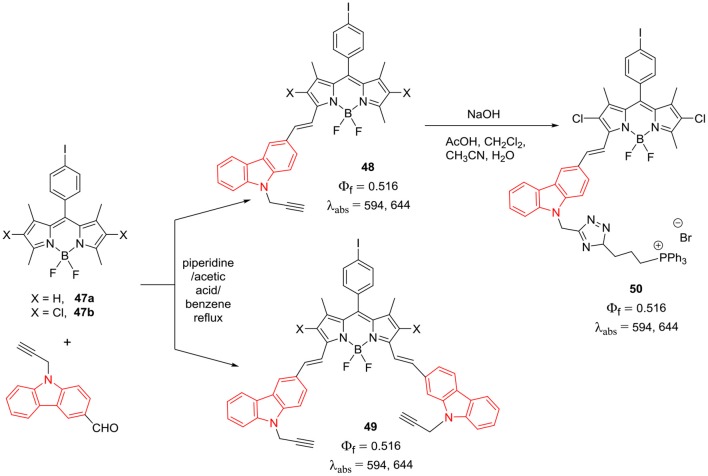
α-styryl linked carbazole-BODIPY based probes for mitochondria imaging.

*Alpha*-styryl substituted BODIPY derivatives have excellent photophysical properties; they exhibit strong absorption and fluorescence in near infra-red (NIR) region. Such α-styryl substituted BODIPY derivatives have tremendous potential as bioimaging agents; particularly cell organelle targeting becomes facile as their absorption and emission falls in the biological window. The α-styryl BODIPYs **48–50** (Scheme 9) having one or two *N*-ethynyl-carbazole groups were prepared by Zhang et al. (2013). Extended conjugation of the BODIPY core with the carbazole ring resulted in strong absorption with high extinction coefficients between 620 and 703 nm and red emission in the range of 650–730 nm. These BODIPYs showed high fluorescence quantum yields and decent two-photon absorption properties; also, the NIR probe **50** demonstrated significant localization in the mitochondria of MCF-7 cells, due to the presence of triphenylphosphonium group (Zhang et al., 2013).

**Scheme 10:**
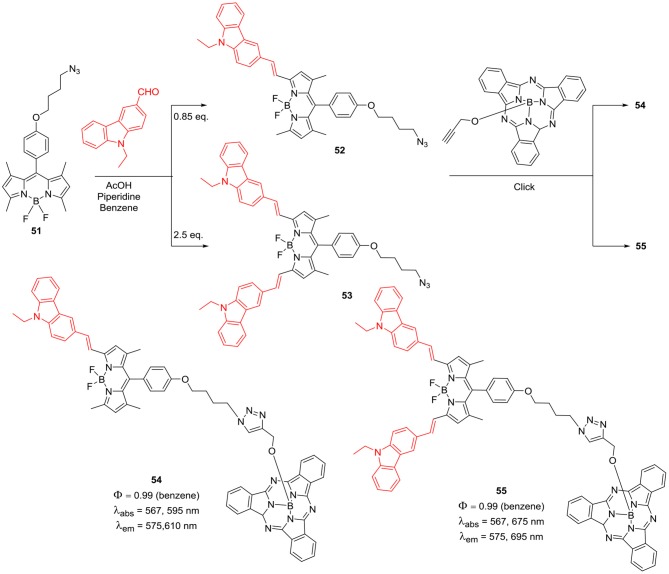
Carbazole substituted BODIPY and subphthalocyanine based light harvesting systems.

An interesting system of subphthalocyanine-BODIPY scaffold containing one or two *N*-ethyl-carbaole moieties was reported by Eçik et al. (2017). Synthesis of these molecules followed multistep synthetic procedure, Knoevenagel condensation of the precursor BODIPY (**51**) with 9-ethyl-9*H*-carbazole-2-carbadehyde (Scheme 10) afforded **52** and **53**. Click reaction of ethyne functionalized subphthalocyanine with BODIPYs **52** and **53**, resulted in the formation of the desired target molecules **54** and **55** (Scheme 10). These systems (**54** and **55**) showed efficient energy transfer from subphthalocyanine unit to the BODIPY unit via fluorescence resonance energy transfer (FRET). Authors suggested that, these kinds of systems can be developed into a BODIPY-based multi-chromophore systems and this will help to reveal their energy transfer potential in efficient light-harvesting systems (Eçik et al., 2017).

**Scheme 11:**
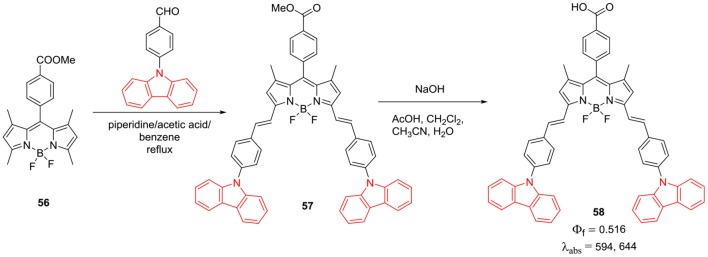
Synthesis of α-Styryl linked *N*-phenyl carbazole BODIPY.

**Scheme 12:**
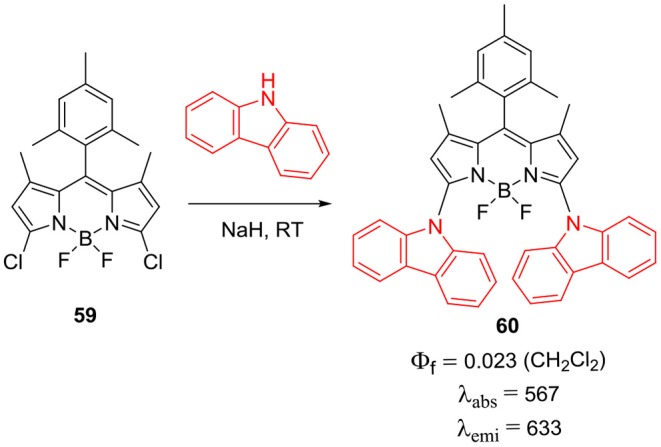
Synthesis of α-*N*-carbazole BODIPY.

Another method to introduce carbazole ring on the BODIPY core is through *N*-linkage (Scheme 11; **57** and **58**). Zhang et al. showed that, 4-(9*H*-carbazol-9-yl)benzaldehyde can be linked to BODIPY **56** via Knoevenagel condensation, followed by the deprotection of **57** to produce **58**. The presence of *N*-phenyl carbazolyl groups on the BODPY **58** can help to reduce the aggregation of dye to some extent; hence **58** exhibited enhanced photon to electron conversion efficiency of 4.4%. Since the electron rich carbazole ring has excellent hole-transport properties; it is widely used in optoelectronics; most of the scientists are interested in substituting carbazole to BODIPY core to enhance their photovoltaic applications (Zhang et al., 2016). A new synthetic approach for the modification of *alpha*-positions of the BODIPY with carbazole was reported by Satoh et al. (2018). As shown in the Scheme 12, to obtain **60**, *alpha*-positions of the BODIPY core can be substituted by nucleophilic aromatic reaction (SNAr) of carbazole with compound **59** in THF (Satoh et al., 2018). Absorption and emission spectra exhibited bathochromic shifts as compared to the parent BODIPY; these molecule promises development of new BODIPY based fluorophores through this synthetic methodology (Satoh et al., 2018).

**Scheme 13:**
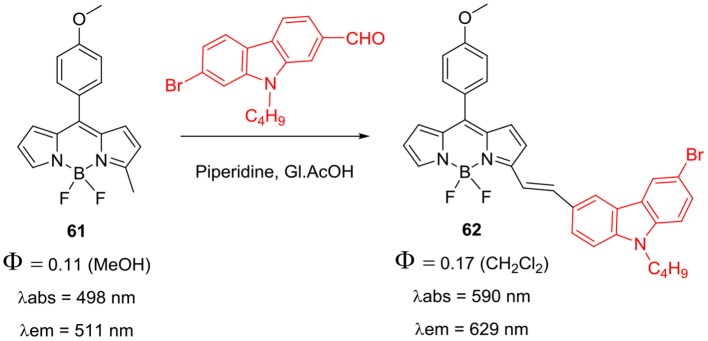
α-Styryl BODIPY with *N*-butylcarbazole group.

**Chart 4:**
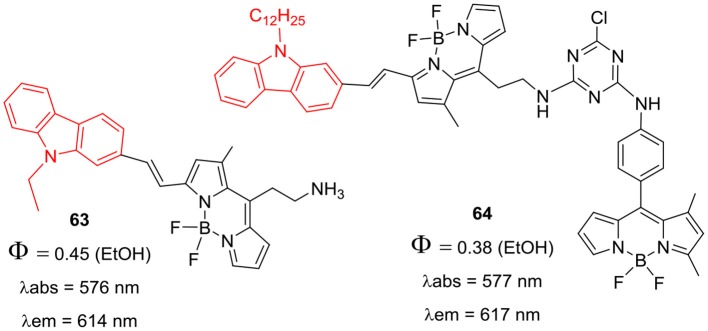
Carbazole-BODIPY based systems for DRET.

*Alpha*-styryl BODIPY having one *N*-alkylcarbazole substituent was prepared by Mani et al. (2017); Knoevenagel condensation of the precursor compound **61** with 7-bromo-9-butyl-9*H*-carbazole-2-carbaldehyde (Scheme 13) afforded **62**. The BODIPY **62** exhibited huge red shifts of 92 nm in the absorption and 118 nm in the emission w.r.t. the starting BODIPY **61** (Mani et al., 2017). Chang et al. reported donor-acceptor systems of *alpha*-styryl BODIPYs **63** and **64** (Chart 4) having *N*-alkylcarbazole and/or cyanuric chloride as linker group (Su et al., 2014b). BODIPYs **63** and **64** exhibited red shifted absorption and fluorescence around 617 nm and considerable pseudo Stokes shift (~120 nm) due to intramolecular DRET (dark resonance energy transfer) in such systems (Su et al., 2014a). Significant pseudo Stokes shifts, red shifted fluorescence, and biocompatibility of cyanuric chloride linker group in **64** suggest its potential application in bioimaging of live cells (Su et al., 2014a).

**Chart 5:**
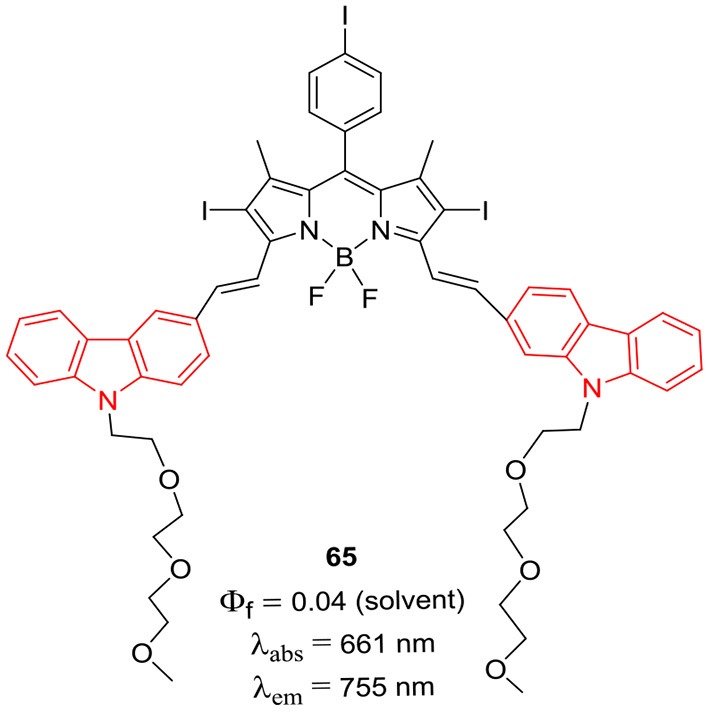
α-Styryl carbazole-BODIPY based nanoparticles for PDT.

**Chart 6:**
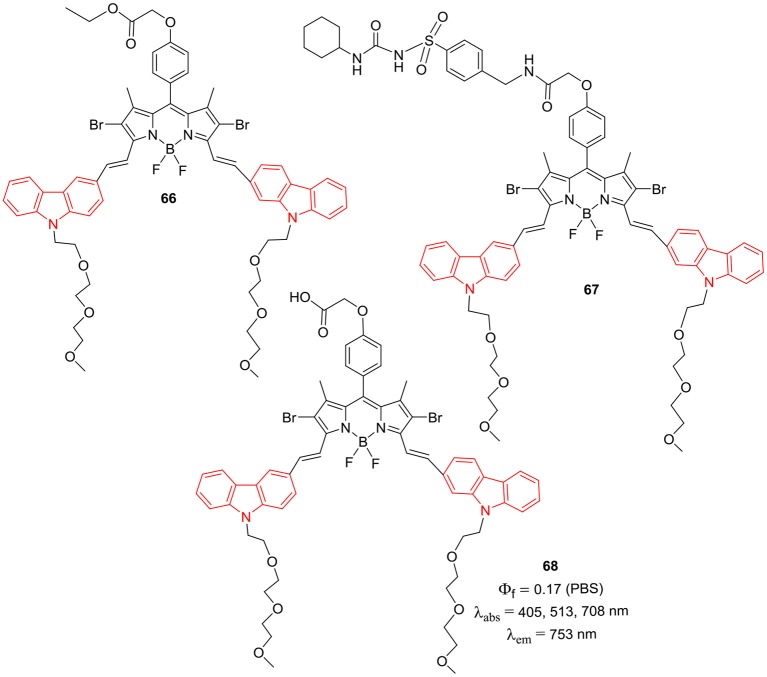
α-Styryl carbazole-BODIPYs for bioimaging and PDT.

Han et al. reported the *alpha*-styryl BODIPY **65** (Chart 5) having two carbazole rings with *N*-PEGylated chains for the deep tissue imaging and PDT application (Huang et al., 2016). The BODIPY **65** showed NIR absorption at 661 and emission at 755 nm with fluorescence quantum yield of 4%. The calculated singlet oxygen quantum efficiency 67% for compound **65** was considerably high and its water soluble nanoparticles were prepared by mixing it with the biodegradable polymer PLA-PEG-FA (comprising of poly-lactic acid, poly-ethyleneglycol, and folate). The biocompatible nanoparticles showed broad absorption and emission in the NIR region (650–800 nm) with 58% singlet oxygen quantum yield upon excitation by NIR light ~670–800 nm; also they can be used for deep tissue imaging and for the treatment of tumors. The organic nanoparticles displayed green emission upon light irradiation in HeLA cells ~670–800 nm and negligible cytotoxicity; making them suitable candidates for PDT studies (Huang et al., 2016). The *alpha*-distyryl BODIPYs **66–68** (Chart 6) having two carbazolylethynyl groups and two *beta*-bromo groups was used as photosensitizers for PDT (Zhou et al., 2018). Compound **67** exhibited strong absorption bands around 513 and 708 nm corresponding to the carbazolyl group and boron-dipyrrin core. For the BODIPY **67** containing glibenclamide analogous moiety, the fluorescene band appeared at 753 with 45 nm Stokes shift; also it demonstrated effective localization in endoplasmic reticulum (ER) of HeLa (human cervical cancer) and HepG2 (human hepatocarcinoma) cells. The compound **67** was able to generate singlet oxygen upon excitation at 610 nm; ER stress was the main reason of cell death as per the report (Zhou et al., 2018).

### *Meso*-Substituted BODIPYs

**Chart 7:**
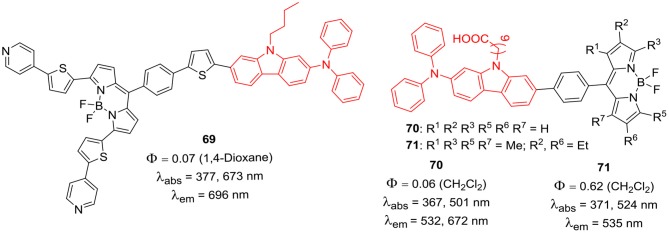
*Meso N*-alkyl carbazole BODIPYs for solar cell application.

Apart from *beta*- and *alpha*- positions of the BODIPY skeleton, the *meso*-position (C-8) can also be substituted with carbazole to prepare variety of dyes with improved absorption and emission properties. Carbazole-based D–π-A type BODIPYs **69–71** were synthesized and studied for DSSC application (Chart 7) (Ooyama et al., 2013a). In BODIPY **69**, two 4-(thiophene-2-yl)pyridine units on the *alpha*-positions served as electron-withdrawing anchor groups and the *meso*-position was substituted with an electron donating 9-butyl-*N,N*-phenyl-7-(thiophen-2-yl)-9*H*-carbazol-2-amine group. The introduction of thiophene linkers between the donor and BODIPY core, extended the π conjugation of the entire system; which was indicated by the noticeable bathochromic shifts in absorption and emission of **69** (673, 696 nm, respectively). In BODIPYs **70** and **71** (Chart 7), the *meso*-substituent: 9-butyl-*N,N*-phenyl-7-(thiophen-2-yl)-9*H*-carbazol-2-amine acted as donor unit, which was linked to BODIPY core with the phenyl linker. The presence of small methyl/ethyl groups on the alpha-position of the BODIPY core in **70** and **71** does not cause much shifts in their absorption and emission maxima (Ooyama et al., 2013b).

The BODIPY **69** showed significant red shift in its absorption and emission; however, it showed moderate performance in DSSC studies. Authors attributed the low performance mainly to the following reasons: (1) Formation of strong π-stacked aggregates of BODIPY on the TiO_2_ surface; (2) The lower LUMO level and the radiation less relaxation of the photoexcited dye which leads to a reduction in the electron-injection yield; (3) faster charge recombination between the injected electrons and ${\text{I}}_{3}^{-}$ ions, leading to a decrease in the *V*_*oc*_ value (Ooyama et al., 2013a). Aggregation between BODIPY cores can be prevented by using co-adsorption of chenodeoxycholic acid (CDCA). One of the main reasons for radiation less relaxation is the free rotation of the aryl substituents at *alpha*- and *meso*-positions of the BODIPY skeleton. This rotation can be reduced by methyl substitution on BODIPY core (Ooyama et al., 2013b). Also, dyes **70** and **71** (Chart 7) showed solid-state red fluorescence and green metallic luster properties in both crystalline and amorphous states (Ooyama et al., 2014).

**Scheme 14:**
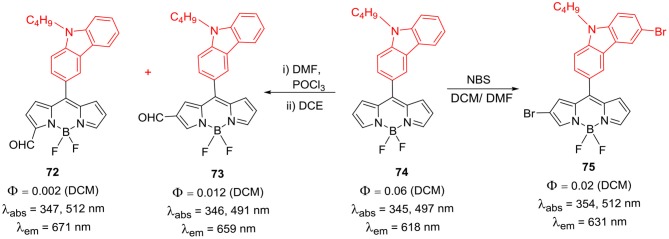
Synthesis of *meso*-carbazole substituted BODIPYs.

**Scheme 15:**
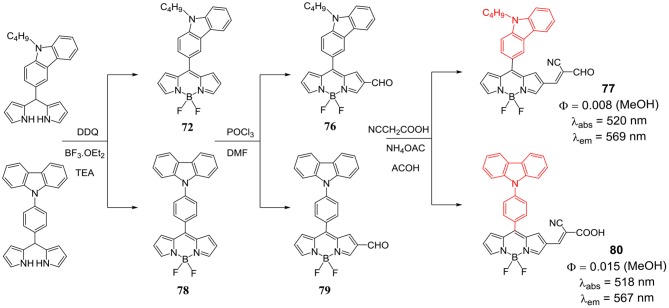
*Meso*-carbazolyl substituted BODIPYs.

Substitution of carbazole unit on the *meso*-position of the BODIPY core through direct linkage is another method to incorporate carbazole unit on BODIPY skeleton. Gupta et al. reported synthesis and photophysical properties of *meso*-substituted carbazole-BODIPY dyad **72** (Scheme 14). This dyad exhibited energy transfer efficiency from donor carbazole unit to the acceptor BODIPY core. As shown in the Scheme 14, dyad was synthesized from the dipyrromethane having *meso*-carbazolyl unit, followed by complexation with BF_3._OEt_2_ to obtain the desired product. BODIPY **72** was further used to make BODIPY derivatives **73–75** (Scheme 14). It was found that the *meso*-carbazoyl group altered the electronic properties of the four BODIPYs which was reflected in the higher extinction coefficient, red-shifted emission maxima, increased quantum yields and large Stokes shifts. Fluorescence studies indicated an efficient energy transfer from *meso*-carbazolyl moiety to the boron-dipyrrin core in all the compounds. Due to increased conjugation with the electron donor *meso*-carbazole group, anodic shifts were observed in the redox potentials of all four BODIPYs **72–75** (Kesavan and Gupta, 2014). The synthesis and photovoltaic application of *meso*-carbazolyl substituted BODIPY based photosensitizers **77** and **80** (Scheme 15) was reported. The photosensitizers were synthesized in four steps from the precursor BODIPYs **72** and **78** as shown in the Scheme 15. The BODIPY **80** exhibited higher photovoltaic performance than the photosensitizer **77** in DSSC studies (Kesavan et al., 2017).

**Chart 8:**
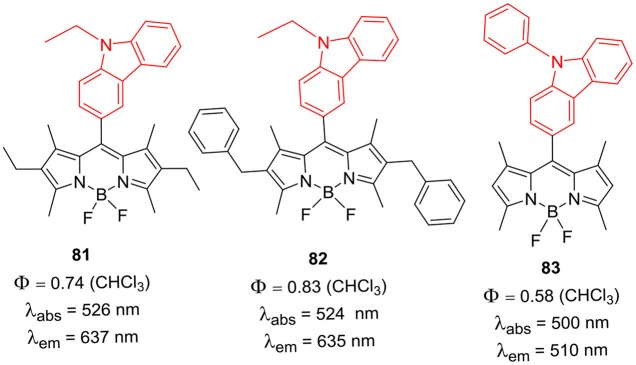
*Meso*-carbazolyl substituted BODIPY derivatives.

**Chart 9:**
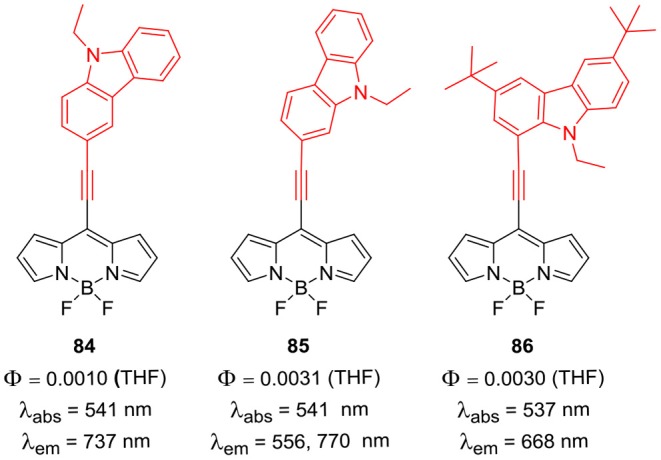
*Meso*-ethynyl linked carbazole-BODIPYs.

Sekar et al. reported BODIPYs **81–83** having 9-ethyl-9*H*-carbazole or 9-phenyl-9*H*-carbazole group at the *meso*-position; the *alpha*- and *beta*-positions were substituted with the alkyl groups (Chart 8). The direct substitution of carbazole ring on the BODIPY skeleton resulted in the enhanced photostability, good lasing ability and singlet oxygen generation property of the dyes (Thorat et al., 2015; Telore et al., 2016). Misra et al. (2014) reported *meso*-ethynyl linked carbazole-BODIPYs **84–86** (Chart 9); efficient intramolecular charge transfer from carbazole unit to the BODIPY unit was observed (Dhokale et al., 2015). Compounds **84–86** showed high open circuit voltage and thus exhibited good application in bulk heterojunction organic solar cells (Jadhav et al., 2015).

**Chart 10:**
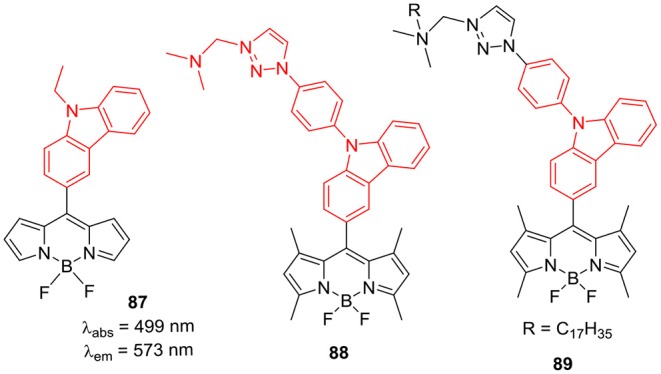
*Meso*-carbazole substituted BODIPYs.

Farfan and Correon-Castro groups (Corona-Sánchez et al., 2016) reported DFT studies of the thin films of *meso*-substituted BODIPY **87** having *N*-ethyl-carabzole ring (Chart 10). The thin films of BODIPY **87** were prepared by the vapor deposition on indium tin oxide; packing morphology of the films was simulated through computational methods and their semi-conductor behavior was predicted. Such kind of DFT study can be helpful when such BODIPY dyes are used in the electronic devices for OPV applications (Corona-Sánchez et al., 2016). Another interesting report by Xing et al., used BODIPYs **88** and **89** to make liposomes encapsulated fluorescent nanoparticles (Lv et al., 2017). The nanoparticles of **88** and **89** showed decent absorption in HEPES buffer at 504 nm; also their emission was centered on 525 nm with about 21 nm Stokes shift. These hydrophilic fluorescent nanoparticles were also tested for live cell imaging on HeLa cells, and the results indicated primary localization in the lysosomes (Lv et al., 2017).

**Chart 11:**
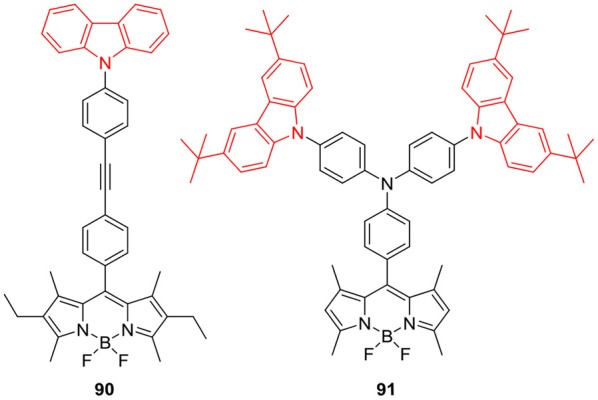
*N*-Phenyl carbazole units linked to *meso*-position of BODIPYs.

**Chart 12:**
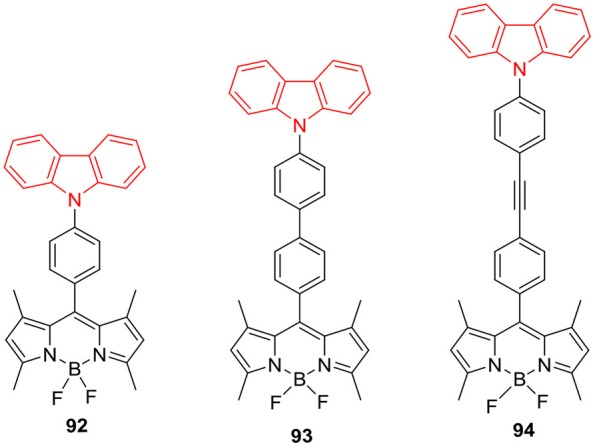
*N*-Phenyl carbazole linked BODIPY with varying linker size.

Carbazole ring can also be linked to the *meso*-position of BODIPY core via *N*-linkage (Chart 11); Nguyen et al. (2014) revealed the OLED application of BODIPY **90**, having 9-phenyl-9*H*-carbazole group at the *meso*-position. BODIPY **90** exhibited green emission with low turn-on voltage in OLED performance, maximum brightness, current efficiency and power efficiency. Report by Li et al. revealed the AIEE ability of BODIPY **91** (Chart 11), which showed enhanced emission in the aggregated form. BODIPY **91** showed weak emission in THF; and the nano aggregates of **91** were prepared in THF water mixture by precipitation method. The noticeable increment in fluorescence intensity of **91** was observed with the gradual increase in the water fraction. In 90% water/THF mixture molecule **91** showed the strongest emission intensity. Similar aggregation study was carried out by preparing carbazole-BODIPY **91** loaded silica nanoparticles, and these nanoparticles demonstrated a stable uniform morphology and strong fluorescence. This AIEE effect was successfully applied for cell imaging and found that BODIPY **91** showed good cellular uptake in MCF-7 cells (Li and Qian, 2016). The recent report by Reddy et al. showed that *meso*-substitution of BODIPY with carbazole ring can be achieved through *N*-linkage; such BODIPYs **92–94** (Chart 12) exhibited photoinduced energy transfer (PEnT). They studied the effect of linker length on PEnT using varying lengths of bridges (phenyl, biphenyl and diphenylethyne) on the BODIPYs **92–94** (Chart 12). Selective excitation of these molecules at carbazole unit resulted in a very efficient energy transfer process (Reddy et al., 2018).

**Chart 13:**
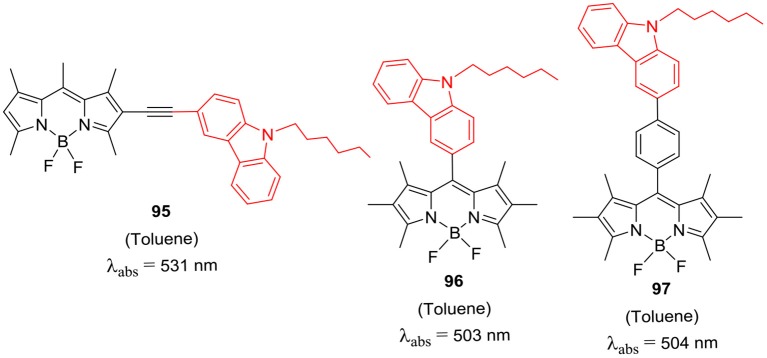
β-substituted and *meso*-substituted carbazole-BODIPYs.

**Scheme 16:**
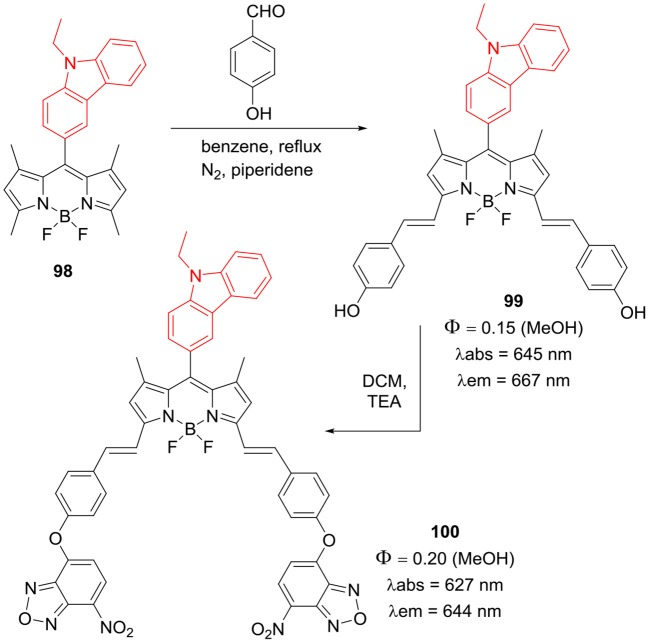
*Meso*-carbazole substituted BODIPY for biothiol detection.

Recently, Thayumanavan et al. prepared donor-acceptor type BODIPYs **95**–**97** (Chart 13) and studied photo-induced electron transfer process for these dyes (Strahan et al., 2019). The electron rich carbazole donor group was attached at either *beta*- or *meso*-position of the BODIPYs to access intramolecular charge transfer (ICT) in the dyes **95**–**97** (Chart 13). The ICT was more facile in the BODIPY **95** as compared to the **96** and **97** in polar solvents; also the carbazole substation at *beta*-position of the BODIPY skeleton shifted the absorption of **95** toward red region with higher molar extinction coefficient than the *meso*- substituted BODIPYs **96** and **97** (Strahan et al., 2019). Qian's group had reported *meso*-*N*-ethylcarbazole substituted BODIPYs **98–100** (Scheme 16); the molecule **101** containing nitro-substituted benzoxadiazole (NBD) moieties, was used as fluorescent probe for biothiol detection and live cell imaging (Xia and Qian, 2018). The probe **100** also demonstrated visible color change from blue to green upon addition of biothiols in the solution; thus it can be used to develop sensor-kit for biothiols in future. In addition, **100** was successfully applied to detect Cys, Hcy, and GSH in living cells (Xia and Qian, 2018).

### Carbazole Bridged BODIPY Dimers

**Chart 14:**
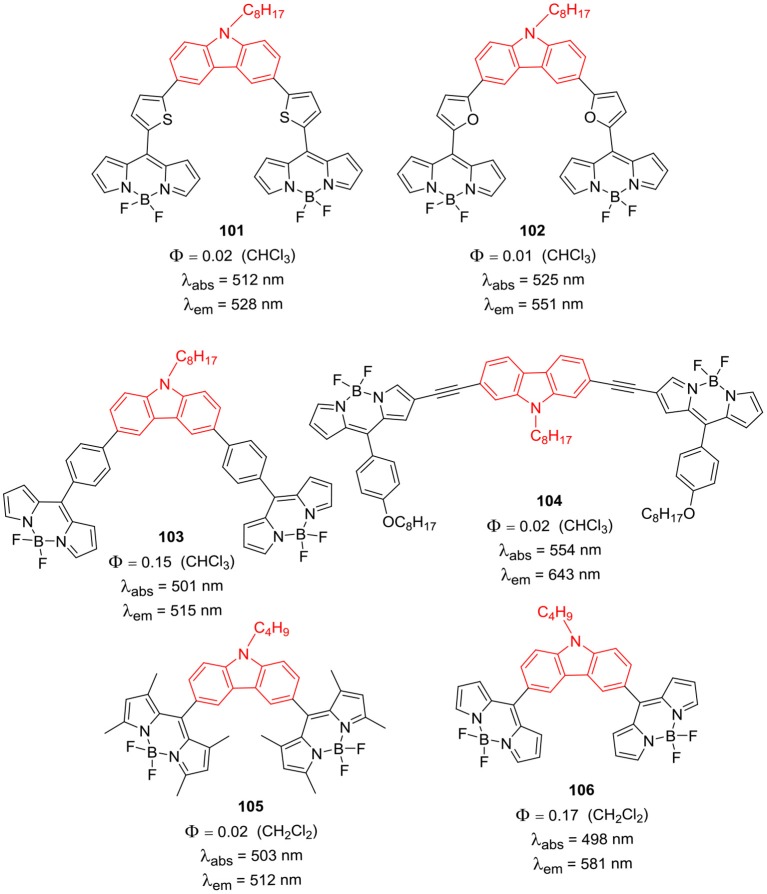
Carbazole bridged BODIPY dimers.

Zong et al. reported carbazole bridged BODIPY dimers **101–103** with extended π conjugation (Chart 14) by introducing linker moieties in between carbazole and BODIPY units. The linkers varied from phenyl, thiophene to furan rings; all these conjugates possess good thermal stability. From the photophysical and electrochemical analysis, it was revealed that thiophene and furan linked carbazole-BODIPY dimers (**101**, **102**) are potential candidates for p-type semiconductor materials in organic solar cells (Zong et al., 2017). Report by Liao et al. showed that presence of alkynyl group as bridging unit (Chart 13; **104**) shifted the absorption and emission toward red region. BODIPY **104** exhibited average photovoltaic performance of 3.1% by a hole mobility mechanism (Liao et al., 2017a). Also, compound **105** (Chart 14) showed decent cytotoxic activity against HT29 cell lines (Sengul et al., 2015). In another report Gupta et al. synthesized *N*-butylcarbazole bridged BODIPY dimer **106** (Chart 14); the compound showed bathochromic shift in the emission band with good Stokes shift of 83 nm (Kesavan et al., 2015).

**Chart 15:**
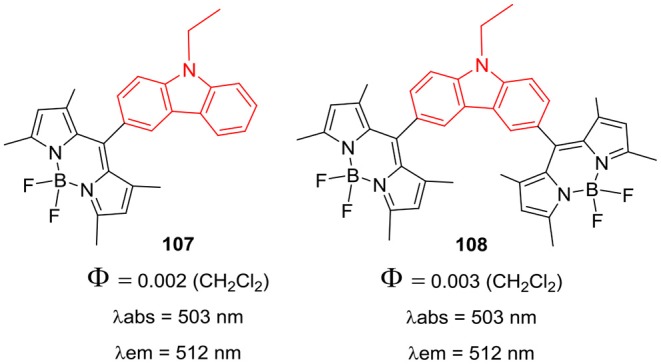
*Meso*-substituted carbazole-BODIPYs.

*Meso*-carbazole substituted BODIPY **107** and N-alkyl-carbazole bridged BODIPY dimer **108** (Chart 15) were prepared and their biological activities were tested in the human colon cancer cell lines (Sengul et al., 2015). The BODIPYs **107** and **108** exhibited strong absorption around 503 nm and fluorescence at 512 nm; cytotoxicity assays in HT29 cancer cells revealed that, **108** is more toxic than **107** (Chart 15). The observed IC_50_ values for **107** and **108** were 21.7 ng/mL and 8.3 ng/mL, respectively (Sengul et al., 2015).

**Chart 16:**
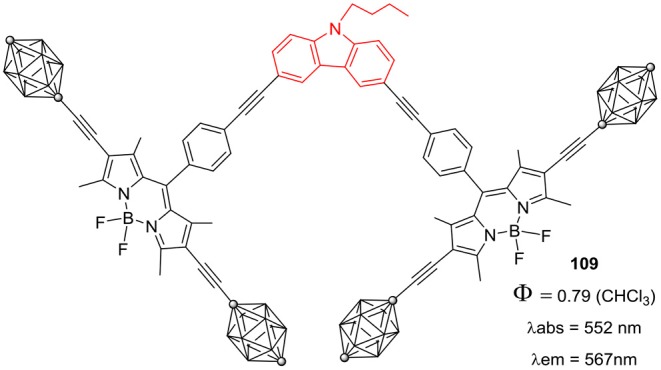
BODIPY based nanocar having *p*-carbone wheels.

BODIPY based nanocar containing *p*-carborane wheels and central *N*-butylcarbazole moiety was prepared by Godoy et al. (2010). The BODIPY nanocar **109** (Chart 16), was highly emissive in nature making it ideal candidate for single molecule fluorescence spectroscopy. Such nanocars are reported to move by an average speed of 4 nm/s on the glass surface under ambient conditions due to the presence of *p*-carborane wheels (Godoy et al., 2010).

### Miscellanious Systems

**Scheme 17:**
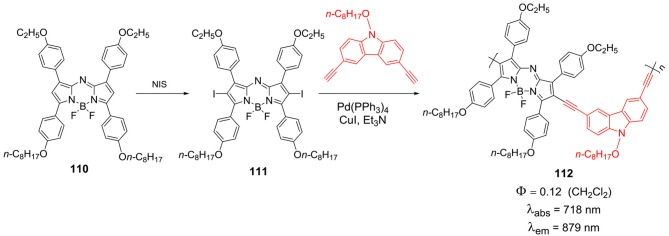
Synthesis of carbazole aza-BODIPY polymer.

In 2013, Ma et al. reported NIR emissive D–π-A polymers **112**, where aza-BODIPY moiety acted as acceptor and electron rich carbazole group served as donor. The synthesis of *beta*-diiodinated derivative of aza-BODIPY **111** was prepared by treating **110** with *N*-iodosuccinimide (Scheme 17). The polymerization of BODIPY **111** was accomplished by a palladium-catalyzed Sonogashira coupling reaction with 3,6-diethynyl 9-octyl-9*H*-carbazole moiety (Scheme 17). The good advantage of such polymer systems is that, these molecules exhibit near-infrared fluorescence around 750 nm and also show tunable band gap in the range of 0.96–1.14 eV. These photophysical and electrochemical properties promises the application of this polymer **112** in device based applications (Ma et al., 2013).

**Chart 17:**
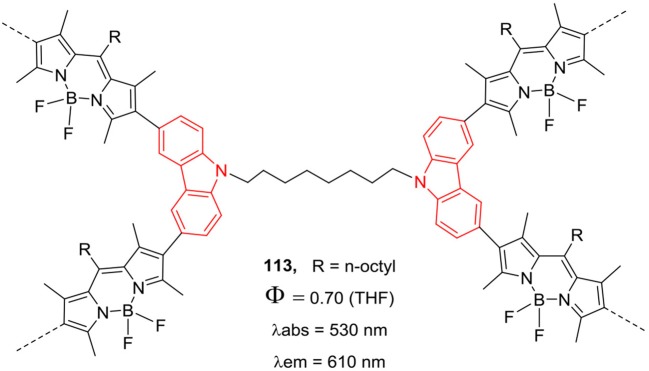
Synthesis of carbazole-BODIPY based soluble polymer.

**Chart 18:**
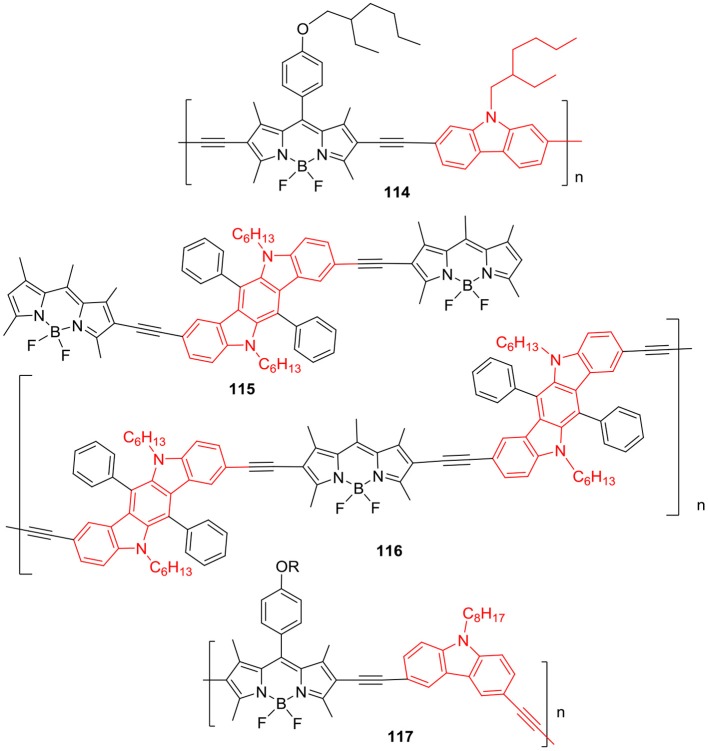
Carbazole-BODIPY based polymers.

Another interesting report by Patra et al. described the synthesis of porous organic polymer **113** (Chart 17) consisting of *N*-alkyl bridged carbazole and BODIPY units (Bandyopadhyay et al., 2018); the macromolecule was able to generate substantial singlet oxygen in solution. The porous soluble polymer **113** exhibited red shifted absorption and emission in the range of 530–610 nm; and used as fluorescent probe for superoxide anion (Bandyopadhyay et al., 2018). The π-conjugated polymers having BODIPY backbone are used as photosensitizers in organic photovoltaics (OPV); and strong absorption in the red or NIR region is desired for high performance of the devices. Combination of BODIPY unit with good electron rich donor moiety in the polymer chain can yield the copolymer with strong absorption profile in the visible-NIR region of the solar spectrum. The optical band gap can be reduced in copolymers by linking donor and acceptor units; Thayumanavan et al. have synthesized π-conjugated BODIPY copolymers **114** (Chart 18) having *N*-alkylcarbazole/dithienopyrrole/bithiophene/fluorene as donor moieties (Popere et al., 2012). The rationally designed copolymers exhibited lower band gap and broad absorption spectra encompassing the entire visible region; thus, making them good panchromatic dyes for OPV applications (Popere et al., 2012). Such copolymers containing π-conjugated donor-acceptor units also show interesting charge transfer and/or energy transfer properties with enhanced absorption and fluorescence in the visible to red region.

Another π-conjugated copolymer **116** incorporating indolo[3,2,-b]-carbazole and BODIPY units (Chart 18) was prepared from **115**, by Khetubol et al. (2015). Copolymer **116** showed broad and red shifted absorption and emission spectra along with the energy transfer from donor indolo[3,2,-b]-carbazole to the acceptor BODIPY unit. The electronic properties of organic π-conjugated polymers can be fine-tuned by introducing electron donor and accepter moieties in the main chain; the resultant macromolecules are popular in OPV, solar cells, and OLEDs, etc. due to light weight and flexible structures. Ma et al. (2014) had reported D-π-A type chiral copolymer **117** by joining BODIPY and *N*-alkylcarbazole via ethyne linkages (Chart 18). Chiral polymer **117** displayed red shifted fluorescence (624–650 nm) and small band gap of about 159–196 eV (Ma et al., 2014). Organic conjugated copolymers are popular for their applications in OLEDs and solar cells due to low cost and light weight; and their electronic properties can be fine-tuned by altering the donor and acceptor units in the polymer backbone. Such BODIPY based macromolecules can be synthesized by replacing the electron donor moieties with different aromatic heterocycles like fluorene, phenothiazine, bithiphene, and carbazole derivatives to enhance their photophysical and electrochemical properties for various applications (Ma et al., 2014).

**Chart 19:**
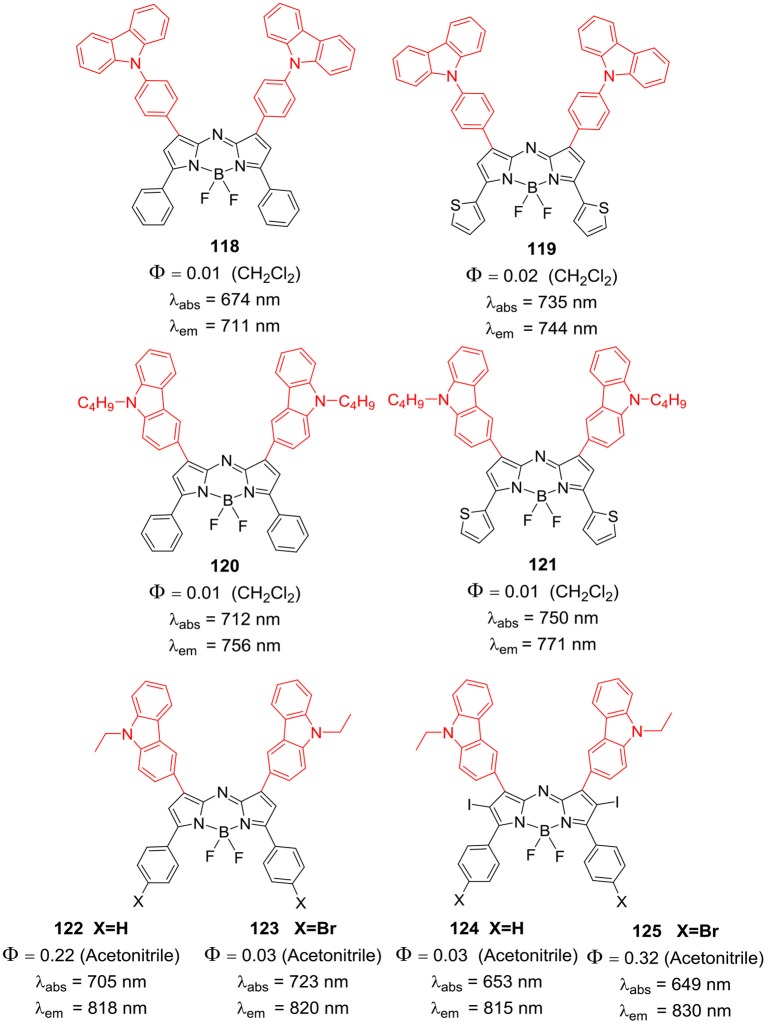
1,7 *N*-phenylcarbazole or *N*-alkylcarbazole substituted aza-BODIPYs.

Aza-BODIPYs are class of BODIPYs which are obtained by substitution of the *meso*-carbon (C-8) atom by nitrogen-atom (Balsukuri et al., 2018). This alteration shifts the absorption and emission maxima of the resultant aza-BODIPY toward NIR region (600–900 nm). Aza-BODIPYs (Balsukuri et al., 2016a) are excellent candidates for the deep tissue imaging as NIR fluorescent probes and as photosensitizers for PDT and DSSC applications. In 2016, Gupta et al. have reported synthesis and optical studies of donor-acceptor type NIR aza-BODIPYs **118–121** (Chart 19), having *N*-phenylcarbazole or *N*-butylcarbazole at the 1,7-positions of the BODIPY core (Balsukuri et al., 2016a). These molecules showed significantly red shifted (~100 nm) absorption and emission relative to the parent tetraphenylaza–BODIPY (Balsukuri et al., 2016b). Also, Fluorescence studies of these molecules suggested effective energy transfer (up to 93%) from donor groups to the aza–BODIPY core. This strategy validated that, simple substitution with energy-donor groups on aza–BODIPYs can induce large red shifts in their electronic spectra, and this approach can be applied to make novel NIR dyes. In 2017, Gawale's group reported synthesis of *N*-ethylcarbazole linked aza-BODIPYs **122–125** (Chart 19), and studied their efficiency to produce triplet excited state and singlet oxygen generation (Gawale et al., 2017). The presence of iodo groups at the *beta*-positions of the aza-BODIPY helped in the enhancement of intersystem crossing efficiency in these molecules. Aza-BODIPYs **122–125** (Chart 19) were able to have sufficiently long triplet excited state and showed 70% singlet oxygen generation efficiency. Also, authors reported application of these molecules in deep tissue photo-acoustic imaging and up to 2 cm deep photoacoustic imaging was successfully demonstrated by using **122** as contrast agent; making it a potential candidate for theranostic application.

**Chart 20:**
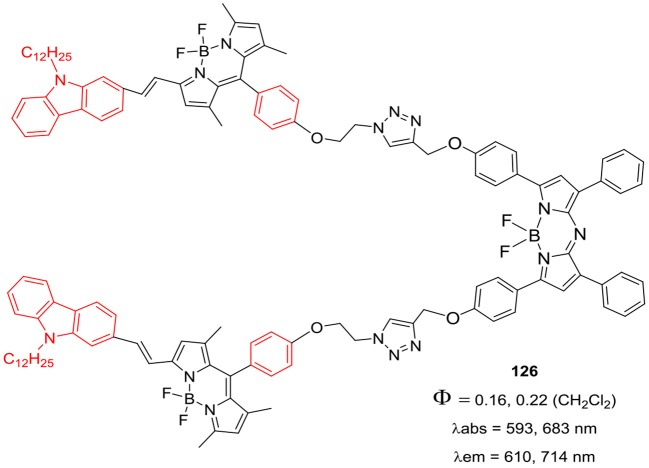
BODIPY triad having Aza-BODIPY as central unit.

**Chart 21:**
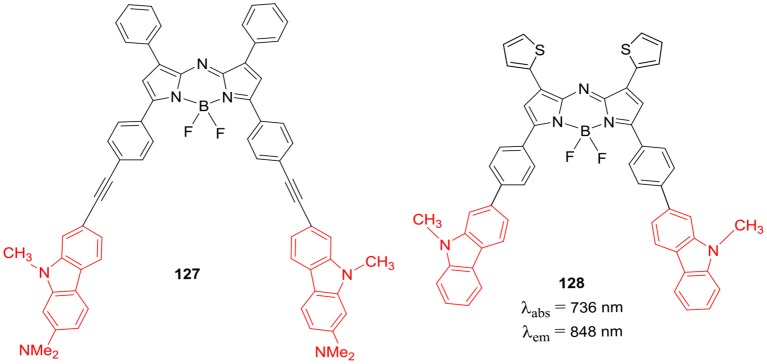
Carbazole linked Aza-BODIPY model system for DFT studies.

Triplet photosensitizers based on porphyrins and transition metal complexes are widely used in photo-catalysis of organic transformations and PDT. However, triplet sensitizers based on BODIPYs have iodo- or bromo- substituents to induce efficient ISC in such molecules. Typically, triplet sensitizers show strong absorption in the visible region, corresponding to the chromophores present in the molecule; though, panchromatic absorption of such molecules is highly desirable for various applications. Zhao and co-workers have combined the BODIPY and aza-BODIPY units to prepare a triad **126** (Chart 20) with interesting photophysical properties (Guo et al., 2014). The triad **126** displayed wide absorption in the visible-red region; along with the intramolecular energy transfer from donor units (BODIPY) and acceptor unit (aza-BODIPY). The resonance energy transfer (RET) in the triad **126** (Chart 20) was helpful to populate triplet excited state upon visible light excitation; which was further used to generate singlet oxygen with 58% quantum yield (Guo et al., 2014). Liu et al. (2013) carried out theoretical calculations for better understanding of the electronic structures and linear absorption of the series of aza-BODIPYs. DFT studies were also performed to investigate the two-photon absorption (TPA) properties of the aza-BODIPYs having various substituents viz. thiophene, phenyl, *N*-alkylcarbazole, fluorine, and pyrene, etc. Among the series of molecules investigated, the aza-BODIPY **127** (Chart 21) with elongated π-conjugated system was predicted to show lower HOMO-LUMO energy gap. Another interesting report by Huang's group discussed the synthesis of color tunable NIR aza-BODIPY **128** (Chart 21) and its application for sensing mercury ions (Liu et al., 2014). The introduction of aromatic carbazole ring induced red-shifts in the absorption and emission spectra; aza-BODIPY **128** (Chart 21) exhibited strong absorption peaks at 720 and 736 nm along with intense fluorescence at 848 nm. The two thienyl groups in the aza-BODIPY **128** served as binding pocket for mercury ion and its fluorescence was quenched after Hg+ binding making it a “turn-off” type fluorescent probe (Liu et al., 2014).

**Chart 22:**
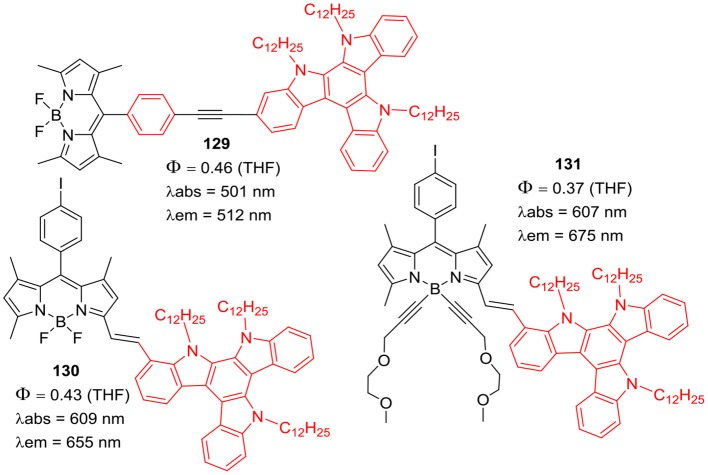
Triazatruxene linked BODIPY dyes.

Ziessel et al. prepared BODIPYs **129–131** (Chart 22) substituted with triazatruxene (TAT) moiety at *alpha*-styryl or *meso*-phenyl positions of the BODIPY skeleton (Bura et al., 2011). TAT is a star shaped molecule consisting of three fused carbazole rings with flat aromatic structure; its derivatives have shown good two-photon absorption (TPA) properties and high hole mobility. The TAT substituted BODIPYs **129–131** exhibited large absorption coefficients and strong emission around 655–675 nm. The interesting electrochemical properties of molecules **129–131** (Chart 22) were examined, these blue dyes exhibited photovoltaic efficiencies in the range of 0.08–0.9% in bulk heterojunction solar cells (Bura et al., 2011).

**Chart 23:**
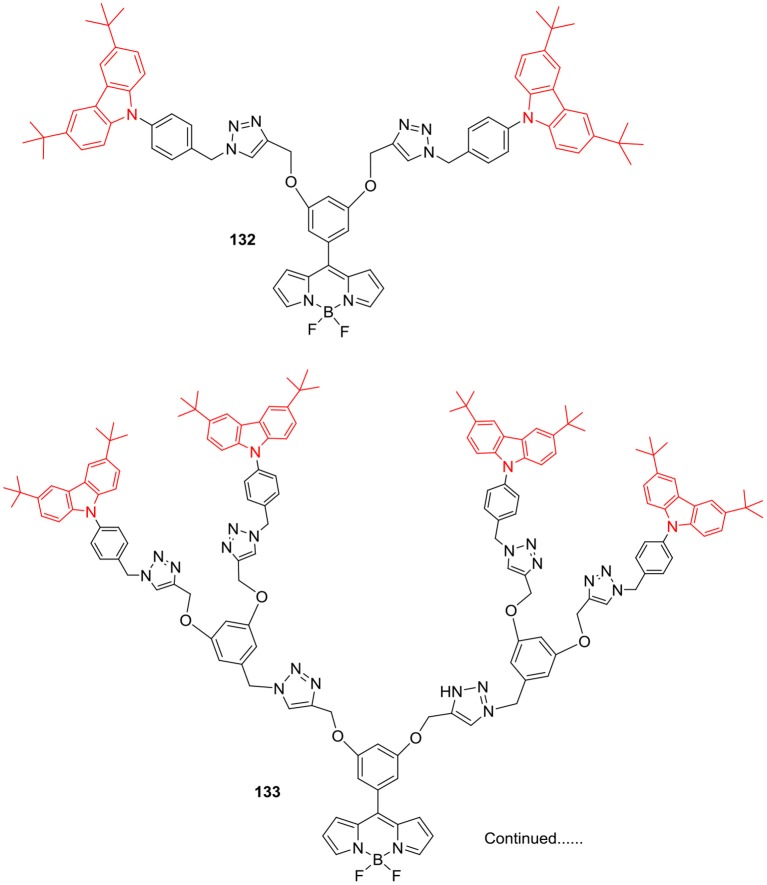
Carbazole based BODIPY core dendrimers (G0, G1 series).

**Chart 24:**
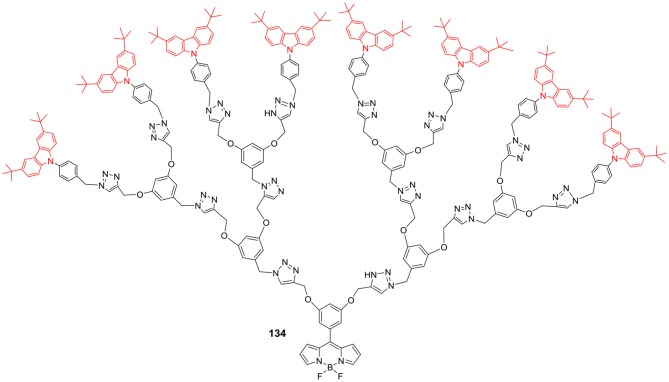
Carbazole based BODIPY core dendrimer, G2 series.

Carbazole is an electron rich aromatic heterocycle and its derivatives are known for their good electronic and hole transport properties; therefore, carbazole conjugated systems have found application in DSSCs and OLEDs. Carbazole based dendrimers can be linked to other chromophores to enhance the absorption and emission properties of such dyes; D-π-A (Donor-π-Acceptor) type BODIPY core dendrimers **132–134** (Chart 23) were synthesized by Babu et al. (2018). The carbazole based dendrimers having BODIPY at the center, displayed rise in their absorption coefficients and red shifted emission upon moving from G0 to G2 generations (Chart 24). Compound **134** (G3 dendrimer) showed 2.7% light to energy conversion as sensitizer in DSSC, which was higher than the G0 and G1 dendrimers. Such carbazole based dendrimers are attractive alternatives for solar light harvesting materials, as compared to small organic molecules (Babu et al., 2018).

**Chart 25:**
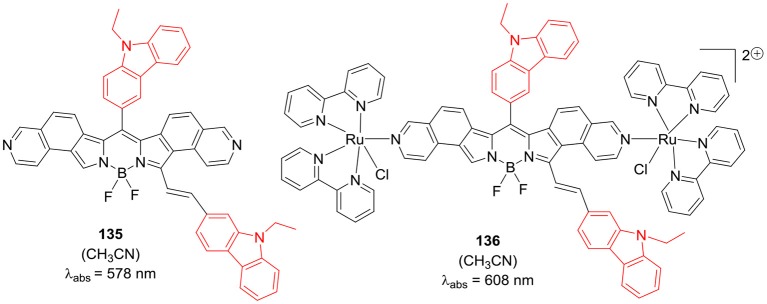
Carbazole -BODIPY bridged Ru(II)polypyridyl complexes.

Typically, BODIPY dyes have short lifetimes for singlet excited states and small Stokes shifts, which restrict their application in solar light harvesting systems. The linking of BODIPY chromophore to another metal complex can overcome such limitation; Chart 25 shows interesting dual emission systems **135** and **136** comprising of carbazole substituted BODIPY unit and Ru(II) polypyridyl unit (Swavey et al., 2019). Compound **135** acted as precursor for the BODIPY and Ru(II)polypyridyl conjugate **136**; the major absorption band was significantly red shifted in the later (Chart 25). Also, the luminescence of the BODIPY unit in **136** was quenched due to the presence of Ru(II) polypyridyl unit; which could be attributed to the increased ISC and other non-radiative decay processes in such conjugates. Compound **136** generated significant singlet oxygen in acetonitrile solution, upon irradiation with long wavelength light; indicating its potential use in PDT (Swavey et al., 2019).

**Chart 26:**
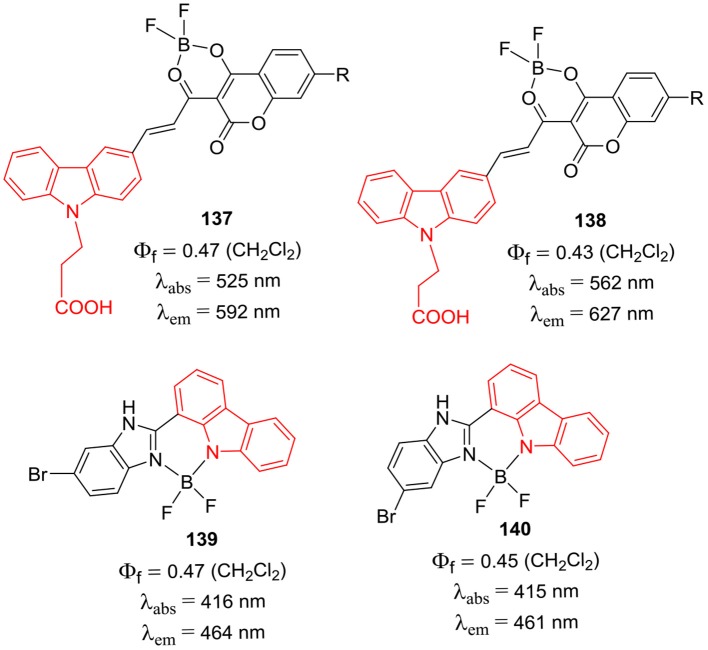
BF_2_ complexes of coumarin-carbazole conjugates (above); BF_2_ complexes of carbazole-benzimidazole conjugates (below).

Sekar et al. have reported coumarin-carbazole conjugates and their BF_2_ complexes **137** and **138** (Chart 26); such D-π-A systems displayed intramolcular charge-transfer process from donor carbazole ring to the coumarin acceptor unit (Rajeshirke et al., 2018). For conjugates **137** and **138**, the emission maxima were observed at 592 and 627 nm, respectively. The strong fluorescence of the BF_2_ complexed coumarin unit in the red region was attributed to the attachment of carbazole donor group; these dyes can be potentially useful for biological applications (Rajeshirke et al., 2018). The BF_2_ complexes of carbazole-benzimidazole conjugates **139** and **140** (Chart 26) were synthesized by Dutta et al. (2017). Both the compounds **139** and **140** displayed relatively large Stokes shifts (34–51 nm) as compared to the typical BODIPY dyes; such molecular scaffolds can be used to develop fluorescence probes in future.

**Chart 27:**
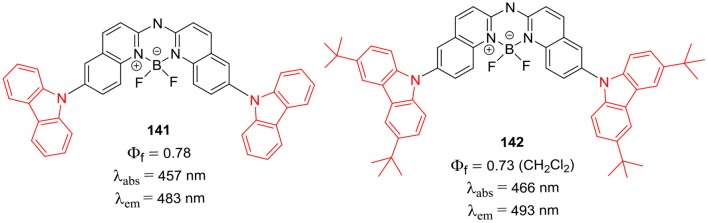
Aza-boron-diquinomethene complexes.

**Chart 28:**
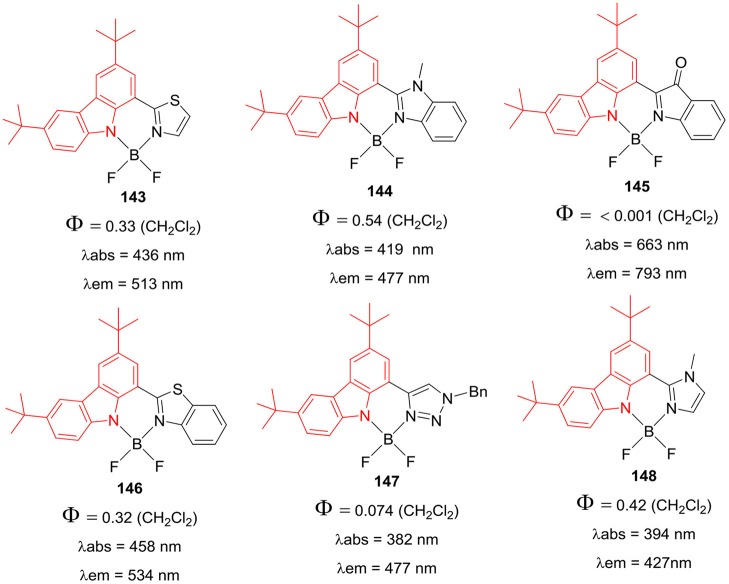
Carbazole based hybrid BODIPYs.

Zhu et al. (2015) have prepared very interesting BF_2_ complexes **141** and **142** (Chart 27); where the aza-dipyrromethene skeleton was replaced by the aza-boron-diquinomethene. The aza-boron-diquinomethene scaffold was substituted with *N*-carbazolyl and/or 3,6-di-tert-butyl-*N*-carbazolyl moieties (Chart 27); the photoluminescence spectra of **141** and **142** showed green-yellow emission due to intramolecular charge transfer. The fluorescence quantum yields were reasonably high between 0.73 and 0.78; such BF_2_ complexes may be suitable for developing pH-sensors and bioimaging probes in future (Zhu et al., 2015). Ema et al. have reported a series of exciting BF_2_ complexes based on carbazole scaffold (Maeda et al., 2016); where carbazole ring was substituted with thiazole, benzothiazole, imidazole, indolone, and benzimidazole (Chart 28). The carbazole-based BF_2_ complexes **143–148** (Chart 28) displayed strong absorption (382–663 nm range) and fluorescence (427–796 nm range) in dichloromethane solution; the emission quantum yield were in the range of 0.074–0.547, except for molecule **145**. The molecules **143**–**148** (Chart 28) exhibited large Stokes shifts of about 76–130 nm in the solutions.

**Chart 29:**
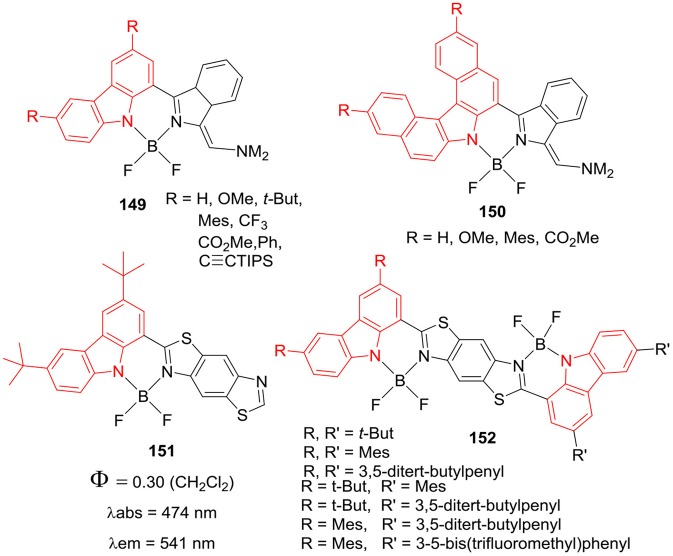
Carbazole based hybrid BODIPYs.

These dyes also showed color tunable solid state emission with emission maxima between 424 and 542 nm range; with the quantum yields around 0.13–0.21. The solid state emission maxima were slightly red shifted relative to those in solution, which was attributed to the J-type packing in the solid state (Maeda et al., 2016). Same group had prepared BF_2_ complexes using organometallic approach, where carbazole was incorporated into the BODIPY framework (Maeda et al., 2015). The substitution of electron withdrawing or electron-donating groups on the carbazole skeleton altered the absorption and emission properties of **149** and **150** (Chart 29). The derivatives of **149** and **150** (Chart 29) showed absorption in the 292–493 nm range and fluorescence maxima between 508 and 650 nm. Also, the derivatives of **149** and **150** showed negligible fluorescence quantum yield with large Stokes shift of 46–142 nm (Maeda et al., 2015).

**Chart 30:**
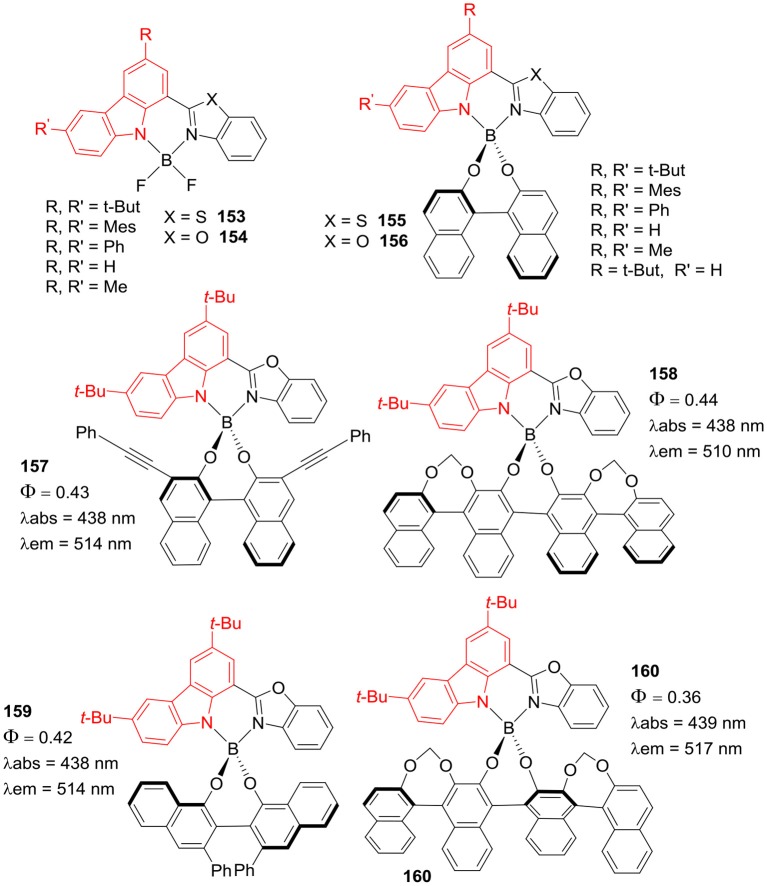
Carbazole based hybrid BODIPYs; data in 1,4-dioxane.

Ema et al. have also reported BF_2_ complexes of carbazole-benzobisthiazole **151** and biscarbazole-benzobisthiazole **152** (Chart 29), these dyes displayed solid state emission in red region (Maeda et al., 2017). The compound **151** exhibited absorption and fluorescence at 474 and 541 nm, respectively; with 40 nm Stokes shift. The derivatives of dimer **152** showed absorption maxima between 516 and 523 nm range and red shifted emission in the range of 547–573 nm, with 31–47 nm Stokes shifts. Interestingly, **151** and the derivatives of **152** (Chart 29) exhibited solid state fluorescence around 564–639 nm, such dyes may have potential application in organic photovoltaics due to strong fluorescence in red-NIR region. Recently, same group has synthesized BF_2_ complexes of carbazole-benzoxazole/carbazole-benzothiazole hybrids **153** and **154**; which were further reacted with binapthyl derivative in the Al-mediated reaction to produce **155** and **156** (Chart 30, Maeda et al., 2019). These chiral dyes **155–160** showed circularly polarize luminescence in solution and in solid state. The major absorption band was centered around 438**–**468 nm and fluorescence maxima were in between 496 and 538 nm; the emission quantum yields were in between 0.22 and 0.44 with considerable Stokes shifts of around 72–75 nm. For compounds **155–160** (Chart 30), the solid state emission bands appeared between 524 and 581 nm; which were red shifted as compared to those in solution. Such chiral BF_2_ complexes of carbazole-benzoxoazole/carbazole-benzothiazole hybrids have potential applications as circularly polarized luminescence materials in the chiral fields (Maeda et al., 2017).

## Summary

Carbazole-containing BODIPYs, carbazole-fused BODIPYs have become quite popular in the recent past; owing to their applications in live cell imaging, light harvesting systems, photovoltaics, and electroluminescent materials. The excellent hole-transport, photorefractive properties, and fluorescent nature of carbazole ring was exploited to design the BODIPY-carbazole conjugates with improved electronic and photovoltaic properties for DSSC and OLED applications. Various synthetic strategies were employed to substitute the three available positions (*alpha, beta* and *meso*) of the BODIPY skeleton; this resulted in the spurt of reports on carbazole substituted BODIPYs. The substitution of electron rich carbazole ring and its derivative on the BODIPY skeleton affected the spectral properties of the parent dye; which reflected in the red shifted absorption and emission maxima of the carbazole-BODIPY conjugates. Typically, direct linkage of carbazole ring on the *alpha*- and *meso*-positions of the BODIPY skeleton caused decent to large Stoke's shifts with fluorescence in the NIR region. Overall, the optical, photophysical, photoluminescent properties of the BODIPY dye can be fine- tuned for the desired application by substituting the carbazole derivatives on the BODIPY core; this knowledge can lead to the development of better more efficient BODIPY dyes in the near future.

## Author Contributions

All authors listed have made a substantial, direct and intellectual contribution to the work, and approved it for publication.

### Conflict of Interest

The authors declare that the research was conducted in the absence of any commercial or financial relationships that could be construed as a potential conflict of interest.

## Abbreviations

AIEE: aggregation induced enhanced emission
BODIPY: difluoroboron dipyrromethene
CDCA: chenodeoxycholic acid
DSSC: dye sensitized solar cell
FET: field effect transistor
FRET: fluorescence resonance energy transfer
ICT: inrtamolecular charge transfer
LHE: light harvesting efficiency
OLED: oraganic light emitting diode
OPV: organic photovoltaic
PET: photoinduced energy transfer
TPEF: two-photon emission fluorescence
TTA: triplet–triplet annihilation.
